# Efficacy of an 8-week course of sofosbuvir and ledipasvir for the treatment of HCV infection in selected HIV-infected patients

**DOI:** 10.12688/f1000research.11397.2

**Published:** 2018-09-21

**Authors:** Onyema Ogbuagu, Ritche Hao, Michael Virata, Merceditas S. Villanueva, Maricar Malinis

**Affiliations:** 1 Department of Medicine, Section of Infectious Diseases, Yale University School of Medicine, New Haven, CT, 06510, USA

**Keywords:** Hepatitis C genotype 1, Direct-acting antivirals, HIV, short-course therapy

## Abstract

**Background:** With the availability of direct acting antiviral treatment for hepatitis C (HCV), HIV and HCV co-infected patients show comparable treatment responses to HCV-monoinfected patients. An 8-week course of sofosbuvir/ledipasvir (SOF/LDV) is highly effective for the treatment of HCV genotype 1 infection in treatment-naïve mono-infected patients with HCV viral loads <6 million IU/ml. There is limited data on the efficacy of this 8-week HCV treatment regimen in HIV-infected individuals with similar viral loads.

**Methods**: The study was a retrospective review of HIV-infected adults coinfected with HCV genotype 1 for whom an 8-week course of SOF/LDV was prescribed by providers at two clinics in the Yale-New Haven Health system from November 1, 2014 until April 30, 2016. Treatment efficacy was assessed as the proportion of treatment initiators who achieved a sustained virologic response 12 weeks after completion of therapy (SVR 12).

**Results:** Nineteen patients met study eligibility criteria and included 14 men (74%); and 12 African-Americans (63%). All patients were on antiretroviral therapy with fully suppressed HIV viral loads and were HCV treatment-naïve. All patients had pre-treatment HCV viral loads <6 million IU/mL. Eighteen patients (95%) completed HCV treatment. Overall, SVR 12 was 95%, with 1 treament failure occurring due to suboptimal adherence.

**Conclusion**: Among our HIV-infected patient cohort with HCV genotype 1 infection, 95% of those treated with an 8 week course of SOF/LDV achieved SVR 12. This is comparable to the efficacy of the same treatment regimen in patients without HIV infection. This study lends proof of concept to the use of shorter course SOF/LDV treatment for HIV-HCV genotype 1 coinfected patients with viral loads <6 million IU/ml. Larger studies are indicated to validate our findings.

## Introduction

HIV and hepatitis C virus (HCV) share similar epidemiologic risk factors and routes of transmission, such that among HIV infected individuals, the prevalence of HCV infection is high and estimated at 25% ( https://www.cdc.gov/hepatitis/populations/hiv.htm). Among certain risk groups, such as injection drug users with HIV infection, prevalence rates as high as 90% have been reported ( https://www.cdc.gov/hepatitis/populations/hiv.htm). HIV infection alters the natural history of HCV disease, such that there are higher and faster rates of progression to liver cirrhosis with its resultant complications; this negative interaction may not be impacted by the receipt of effective antiretroviral therapy (ART) ^1, 2^.

Since the sequencing of the HCV genome, there has been an explosion in the number of drugs approved for the treatment of HCV infection. The direct acting antiviral treatment regimens now allow for fully orally administered, well tolerated, and highly effective treatments for HCV infection. However, multiple studies have shown that of the individuals eligible for HCV treatment, few have received treatment ^3, 4^. A primary obstacle to the treatment of HCV infection is the exorbitant cost of the treatment regimens ^5, 6^. Therefore, cost saving measures including shortened duration of therapy are of interest to patients and their providers ^7^. Emerging data suggests that 8-week rather than 12-week regimens may be effective for HCV treatment among selected patients ^8, 9^.

The United States Food and Drug Administration (FDA) approved sofosbuvir/ledipasvir (SOF/LDV) in 2014 for the treatment of chronic HCV genotype 1 infection. The ION-3 study of SOF/LDV that included treatment-naïve non-cirrhotic patients with HCV genotype 1, found that the sustained virologic response 12 weeks after end of therapy (SVR12) was comparable between the 8-week (with and without ribavirin) and 12-week treatment arms in a post hoc analysis for patients who had a pre-treatment HCV viral load (VL) <6 million IU/ml ^10^. Based on this data, the American Association for the Study of Liver Diseases/Infectious Diseases Society of America guidelines recommend that treatment-naïve, genotype 1 patients without cirrhosis, who are non-black, HIV-negative and with a pre-treatment HCV VL <6 million IU/ml ( http://www.hcvguidelines.org/full-report/hcv-testing-and-linkage-care) can be successfully treated with 8 weeks of SOF/LDV. However, the guidelines cite limited data as the reason to not recommend an 8-week SOF/LDV treatment course for HIV-infected patients.

Contemporary HCV treatment trials with directly acting antiviral (DAA) agents have shown that HIV infection status no longer independently impacts treatment outcomes ^11, 12^. Therefore, shorter HCV treatment regimens are likely to be as effective in HIV-infected individuals as their non-infected counterparts. Our study describes treatment outcomes of a short (8 week) course of SOF/LDV in HIV/HCV co-infected patients.

## Methods

We performed a retrospective review of all HIV and HCV co-infected patients, for whom an 8-week SOF/LDV treatment course was initiated from November 1, 2014 until April 30, 2016. The treatment decision for short course therapy was made by individual clinic providers at two clinics based at Yale Health system in New Haven, CT, USA: Nathan Smith Clinic and the Haelen Center.

Eligibility criteria for the study included all adult (age >18 years) patients with confirmed HIV infection who had HCV genotype 1 infection. Only individuals for whom treatment with an 8-week course of SOF/LDV was intended, were included in the analysis.

Electronic medical records of eligible patients were reviewed. Data collected included demographics, HIV clinical data (CD4 count, HIV VL, antiretroviral treatment (ART)), and laboratory data, including complete blood counts, electrolytes, and liver function tests and biopsy results. Plasma HCV viral loads and genotypes were determined at our lab using COBAS Ampliprep/COBAS Taqman HCV Test, v2.0 (Roche Diagnostics, Indianapolis, IN, USA). Assessment of liver fibrosis stage at time of treatment initiation was determined by one or more of the following: liver biopsy and non-invasive liver fibrosis scores, such as AST to platelet ratio index (APRI) ^13^ and fibrosis-4 (FIB-4) score ^14^. Patient-reported adverse events and reasons for non-completion or discontinuation of treatment were based on documentation in electronic medical records. Data were recorded and analyzed using descriptive statistics in Microsoft Excel, v2013. Overall SVR 12 rate was defined as the proportion of individuals for whom an 8-week treatment course was initiated that had undetectable HCV viral loads 12 weeks after completion of therapy.

Study approval was obtained from the Yale University Human Investigations Committee (number 1506016104).

## Results

A total of 19 patients met the study inclusion criteria. Median age was 53 years (IQR 42-73 years); 14 (74%) were males, and 12 (63%) were African-American. The median body mass index was 28.2 kg/m2. The majority (95%) had glomerular filtration rate >60 ml/min. The major risk factor for HIV was injection drug use (53%). Median CD4 T cell count was 678 cells/µL (IQR 458-1004 cells/µL). All patients were on ART, of which non-nucleoside reverse transcriptase inhibitors (43%) followed by integrase strand transfer inhibitor-based regimens (32%) were most common. Thirteen patients (68%) were on tenofovir/emtricitabine (FTC) and 5 (26%) were taking abacavir/lamivudine (3TC). Patients who were on HIV protease inhibitors were receiving tenofovir/FTC. All patients had fully suppressed HIV VLs ( Table 1).

**Table 1.** Baseline demographic and clinical characteristics of 19 HIV and HCV co-infected patients treated with an 8 week course of sofosbuvir and ledipasvir.

Characteristic	Value
Age, median (years; median, IQR)	53 (49.5-60.0)
Gender (male; n, %)	14 (74)
Race (n, %) African-American Caucasian	12 (63) 7 (37)
BMI (kg/m ^2^; median, IQR)	28.2 (24.5-29.7)
Creatinine clearance (ml/min; n, %) <60 >60	1 (5) 18 (95)
HIV risk factor (n, %) IDU Heterosexual contact MSM Blood transfusion	10 (53) 6 (31) 2 (11) 1 (5)
CD4 count (cells/µL; median, IQR)	678 (458-1004)
ART regimen (n, %) NNRTI PI INSTI Other	8 (42) 4 (21) 6 (32) 1 (5)
ART regimen: NRTI component (n, %) Tenofovir/FTC Abacavir/3TC	13 (68) 5 (26)
HCV genotype (n, %) 1a 1b 1 unspecified	12 (63) 5 (26) 2 (11)
HCV viral load (IU/ml; median, IQR)	869,000 (275,500-1,925,000)
Baseline LFTs (U/L; median, IQR) ALT AST	45 (30-70) 39 (30.5-62.5)
APRI score (n, %) < 0.7 0.7- < 1.0 > 1.0	10 (53) 4 (21) 5 (26)
FIB 4-score (n, %) <1.45 1.45- < 3.25 > 3.25	5 (26) 12 (63) 2 (11)

ALT, alanine transaminase; APRI, AST to platelet ratio index; ART, antiretroviral; AST, aspartate transaminase; BMI, basal metabolic index; FIB-4, fibrosis 4; HCV, hepatitis C virus; HIV, human immunodeficiency virus; IDU, injection drug use; IQR, interquartile range; IU/ml, international units/milliliter; LFT, liver function test; MSM, man who has sex with men; NRTI, nucleoside(tide) reverse transcriptase inhibitor, NNRTI, non-nucleoside reverse transcriptase inhibitor; PI, protease inhibitor; INSTI, integrase strand transferase inhibitor; U/L, units/litre; FTC, emtricitabine; 3TC, lamivudine.

Twelve (63%) patients had HCV genotype 1a and 5 (26%) had genotype 1b; in 2 patients, genotype 1 subtype was not done. Median AST and ALT values were 39 (IQR 31-63) units/L and 45 (IQR 32-70) units/L, respectively. All patients had baseline HCV VLs of < 6 million IU/mL and were HCV DAA treatment-naïve. Based on concordant non-invasive scoring (APRI and FIB-4 score), we classified two patients as having cirrhosis, (patients 2 and 3 in database), but both were clinically compensated (Child-Pugh Class A). One patient (patient 15) had discordant APRI (above cut-off) and FIB-4 scores (below cut-off), so was not classified as having cirrhosis.

Eighteen patients (95%) completed 8 weeks of therapy. One patient was non-adherent due to active substance abuse and only completed the first 4 weeks of treatment. Adverse events while on treatment were reported by 6 patients as follows: diarrhea (n=1), abdominal pain (n=1), nausea (n=1), poor appetite (n=1), diffuse joint pains (n=1), and pruritus without rash (n=1). One patient who experienced fatigue due to influenza temporarily discontinued treatment for 7 days, but resumed treatment afterwards. There were no cases of renal insufficiency, including patients who were on HIV protease inhibitors and tenofovir/FTC. Overall, SOF/LDV was well tolerated with no treatment discontinuations due to adverse effects.

All eligible patients had at least one HCV VL assay performed either at week 4 or week 8 of treatment and at 12 weeks following completion of therapy. At week 4 of treatment, 11 of 12 patients for whom there was available data, had undetectable HCV VLs; one patient had viremia that was less than the lower limit of detection of the assay (< 15 IU/ ml). At week 8 of treatment, 11 of 12 patients, who had available HCV VLs, had undetectable HCV VLs. The patient who had detectable HCV VL at week 8 had completed only 4 weeks of therapy and was subsequently non-adherent due to active substance use. In total 18 of the 19 patients achieved SVR 12. Therefore, overall SVR 12 rate was 95% ( Table 2). The two patients who had cirrhosis also achieved SVR 12.

**Table 2.** Treatment outcomes in 19 HIV and HCV co-infected patients treated with sofosbuvir and ledipasvir.

	4 week HCV VL n=12 ^1^	8 week HCV VL n=12 ^2^	SVR 12 n=19 ^3^
Undetectable HCV VL (n, %)	11 (92)	11 (92)	18 (95)

HIV, human immunodeficiency virus; HCV, hepatitis C virus; SVR 12, sustained virologic response 12 weeks after completion of therapy; VL, viral load. ^1^Data only available for 12 patients; ^2^Data only available for 12 patients (1 patient only completed 4 weeks of treatment); ^3^Data obtained from 19 patients 12 weeks after completion of treatment.

Spreadsheet data showing baseline demographic and clinical characteristics, as well as treatment outcomes, of HIV-HCV patients treated with 8-week course of sofosbuvir/ledipasvir Click here for additional data file. Copyright: © 2018 Ogbuagu O et al. 2018 Data associated with the article are available under the terms of the Creative Commons Zero "No rights reserved" data waiver (CC0 1.0 Public domain dedication).

## Discussion

HIV and HCV infections are often referred to as syndemics as they share similar routes of transmission and impact populations that have similar demographic and socio-economic profiles ^15^. The presence of HIV infection confers a risk for accelerated progression of liver disease, even when HIV is virally suppressed ^16^. For these reasons, it is important to ensure treatment of HCV for all HIV-infected persons, regardless of disease stage. Multiple studies have shown that HIV co-infection is no longer a significant predictor of poor HCV treatment outcomes, such that cure rates among individuals infected with HIV are similar to those who are uninfected ^11, 17^.

There is interest in shorter HCV treatment durations for a number of reasons: the prohibitive cost of newer DAAs ^18^, and issues of adherence and potential development of resistance or toxicity. Kowdley *et al.* showed in a post hoc analysis that an 8-week treatment regimen of SOF/LDV resulted in a high SVR 12 rate among non-cirrhotic HCV infected individuals with genotype 1 infection that was non-inferior to an 8-week regimen with ribavirin or a 12-week regimen without ribavirin ^10^. Lower relapse rates were observed among patients receiving 8 weeks of SOF/LDV who had baseline HCV RNA levels <6 million IU/ml (2%; 2 of 123). However, this study did not include HIV-infected individuals.

In a subsequent real-world multi-national retrospective study of 634 patients, an 8-week course of SOF/LDV resulted in an overall SVR 12 of 98.1% in non-cirrhotic treatment-naïve individuals regardless of HCV VLs. This study included 16 HIV-infected individuals, and for those with VL >6 million IU/ml, 100% achieved SVR 12 ^9^. Unlike the previous study, this study found that pre-treatment HCV VL >6 million IU/ml in a subset of patients with HIV infection did not affect treatment outcomes, including relapse rates.

Our study, showing an SVR 12 of 95%, is similar to the rates observed in a German cohort of 28 HIV-HCV co-infected patients, who showed a 96% response rate to 8 week therapy using SOF/LDV (GECCO-01 study) ^8^. All patients in the trial were on antiretroviral therapy with a median CD4 count of 604 cells/mm ^3^. However, the cohort consisted of predominantly Caucasian and male patients (89%). Our patient demographic was different with more women (26% versus 11%), and comprised a majority of African-American patients (63%).

It is important to highlight that not all DAA-based 8 week treatment courses for HIV-infected patients have yielded satisfactory results. The phase 3 ALLY-2 study, explored 8-week and 12-week SOF/daclatasvir treatment courses in HIV-infected individuals with HCV genotypes 1-4 ^19^. For treatment-naïve patients with HCV genotype 1, SVR 12 was 96% in the 12-week arm and 76% in the 8-week arm. However, it was observed that patients with HCV VL <2 million IU/ml performed better than those with viral loads >2 million IU/ml (SVR 12 of 100% versus 62%) supporting excellent efficacy with lower viral loads ^19^.

Our 95% SVR 12 rate in individuals placed on short course treatment may be attributable to certain factors: excellent adherence (supported by well controlled HIV infection) and selection of individuals with low HCV viral loads, factors that are associated with higher likelihood of cure ^17, 20^. It is remarkable that 26% of subjects were women and almost two-thirds were African-American; two groups that are often under-represented in HCV treatment studies, and this thereby increases the generalizability of the study results. The two individuals with cirrhosis also achieved excellent treatment results. In spite of the small number of patients in our study, the concordance of our findings with the European cohort in the GECCO-01 trial, as well as the multi-center study reported by Kowdley *et al*, lends support to its validity.

A limitation of our study is that it was retrospective, therefore data captured was dependent on the quality of documentation by patient providers. Our patient demographic may not be representative of patients in settings different from ours. There may be a treatment selection bias, whereby patients who were more likely to adhere to therapy and had characteristics favorable to achieving an optimal response were initiated on therapy by their clinic providers. Due to the low number of patients with cirrhosis in our cohort, it is not advisable to extend the conclusions to this subgroup.

In summary, our study provides support for the use of an 8-week course of SOF/LDV as an effective treatment option for HIV and genotype 1 HCV co-infected individuals with HCV viral loads <6 million IU/ml.

## Data availability

The data referenced by this article are under copyright with the following copyright statement: Copyright: © 2018 Ogbuagu O et al.

Data associated with the article are available under the terms of the Creative Commons Zero "No rights reserved" data waiver (CC0 1.0 Public domain dedication). 

Dataset 1: Spreadsheet data showing baseline demographic and clinical characteristics, as well as treatment outcomes, of HIV-HCV patients treated with 8-week course of sofosbuvir/ledipasvir. doi, 10.5256/f1000research.11397.d218522 ^21^

## Ethical statement

This medical review was approved by Yale University Human Investigations Committee (number 1506016104); individual patient consent was not required in this retrospective chart review.
