# Supplementary material for: Efficacy of an 8-week course of sofosbuvir and ledipasvir for the treatment of HCV infection in selected HIV-infected patients
Source: F1000Res. 2018 Sep 21;6:620. Originally published 2017 May 3. [Version 2] doi: 10.12688/f1000research.11397.2 (PMC6171717; doi:10.12688/f1000research.11397.2)
Supplement: Peer review history

# Peer review: Referee response for version 2

**DOI:** 10.5256/f1000research.17847.r38623

**Published:** 2018-09-24

**Authors:** David K Wong

**Affiliations:** 1 Immunodeficiency Clinic, University Health Network, Toronto, ON, Canada; 2 University of Toronto, Toronto, Canada

Revision is fine.

I have read this submission. I believe that I have an appropriate level of expertise to confirm that it is of an acceptable scientific standard.

# Peer review: Referee response for version 1

**DOI:** 10.5256/f1000research.12302.r24844

**Published:** 2018-08-30

**Authors:** David K Wong

**Affiliations:** 1 Immunodeficiency Clinic, University Health Network, Toronto, ON, Canada; 2 University of Toronto, Toronto, Canada

General:

 This is a small retrospective study that adds to the evidence that those with HCV (genotype 1)-HIV co-infection and low viral load can be treated successfully with 8 weeks of Sofosbuvir/Ledipasvir. Adherence matters as the one treatment failure did not complete treatment. The data from this cohort (two patients not described) are not strong enough to recommend this strategy for those with established cirrhosis. On-treatment monitoring of HCV PCR adds little to treatment.

 Specific comments:

 1. The introduction is a bit dated. We should no longer need to justify treatment of those with HIV co-infection as a priority population. The simple fact of HCV infection means that these individuals should be offered treatment.

 2. Introduction points out that HIV infection status no longer independently impacts treatment outcomes. The introduction should ALSO point out that those with HCV without HIV, NO cirrhosis and low viral load can be successfully treated with 8 weeks of SOF/LDV.

 3. Two patients were thought to be cirrhotic but clinically compensated - how compensated? They should be described further and they should be pointed out in Table 3 - what were platelet counts, INR, albumin, Bilirubin.

 4. The discussion is repetitive - should not repeat what was said in introduction

 5. Study presents data of on-treatment HCV PCR monitoring. Do the authors think that this is required?

I have read this submission. I believe that I have an appropriate level of expertise to confirm that it is of an acceptable scientific standard, however I have significant reservations, as outlined above.

# Response

**Published:** 2018-09-14

**Authors:** Merceditas Villanueva

**Affiliations:** Yale University, USA

General:

This is a small retrospective study that adds to the evidence that those with HCV (genotype 1)-HIV co-infection and low viral load can be treated successfully with 8 weeks of Sofosbuvir/Ledipasvir. Adherence matters as the one treatment failure did not complete treatment. The data from this cohort (two patients not described) are not strong enough to recommend this strategy for those with established cirrhosis. On-treatment monitoring of HCV PCR adds little to treatment.

*Response- Thanks for the comment.*

*Regarding the concern about recommending short course treatment for cirrhotics, we acknowledge in the limitations section that… “* *Due to the low number of patients with cirrhosis in our cohort, it is not advisable to extend the conclusions to this subgroup.”*

*Also, we agree that on-treatment monitoring of HCV viral loads adds little to treatment especially as there are no “stoppage” rules based on pre-set viral load decay parameters monitored over time. We captured HCV viral load assessments as checked by clinic providers which was based on the clinic HCV treatment protocol at the time the study was conducted.*

Specific comments:

1. The introduction is a bit dated. We should no longer need to justify treatment of those with HIV co-infection as a priority population. The simple fact of HCV infection means that these individuals should be offered treatment.

*Response- we agree that there is no longer a need to justify treatment of HIV infected patients with HCV as they experience similar treatment outcomes. We have eliminated the sentence “owing to this the treatment of HCV infection is prioritized for persons infected with HIV” from the revised manuscript*

2. Introduction points out that HIV infection status no longer independently impacts treatment outcomes. The introduction should ALSO point out that those with HCV without HIV, NO cirrhosis and low viral load can be successfully treated with 8 weeks of SOF/LDV.

*Response: We have modified the 3 ^rd^sentence of paragraph 3 of the introduction as follows: “* *Based on this data, the American Association for the Study of Liver Diseases/Infectious Diseases Society of America guidelines recommend that  treatment-naïve, genotype 1 patients without cirrhosis, are non-black, HIV-negative and with a pre-treatment HCV VL <6 million IU/ml ( http://www.hcvguidelines.org/full-report/hcv-testing-and-linkage-care) can be successfully treated with 8 weeks of SOF/LDV.*

3. Two patients were thought to be cirrhotic but clinically compensated - how compensated? They should be described further and they should be pointed out in Table 3 - what were platelet counts, INR, albumin, Bilirubin.

*Response: We noted discrepancies in the dataset attached to our original submission when compared to our original data! (the APRI and Fib-4 scores were erroneously arranged in descending order on the submitted version and not on the same row with appropriate patients). We have corrected this on the re-submission.  Both patients had APRI and Fib-4 scores that were above cut-off values that are suggestive of liver cirrhosis. We used the term “compensated” to mean that they had no documented clinical features of decompensation including development of HCC, ascites, porto-systemic encephalopathy or varices. We did calculate Child Pugh scores (inclusive of INR and albumin levels) and both patients were class A. Both of these last points are mentioned in the results section.*

4. The discussion is repetitive - should not repeat what was said in introduction

*Response: Thank you. We have modified the discussion to remove redundant / repetitive statements.*

5. Study presents data of on-treatment HCV PCR monitoring. Do the authors think that this is required?

*Response: as stated in our first response to general comments -* *we agree that on-treatment monitoring of HCV viral loads adds little to treatment especially as there are no “stoppage” rules based on pre-set viral load decay parameters monitored over time. We captured HCV viral load assessments as checked by clinic providers which was based on the clinic HCV treatment protocol at the time the study was conducted*

# Peer review: Referee response for version 1

**DOI:** 10.5256/f1000research.12302.r22470

**Published:** 2017-06-05

**Authors:** Patrick Ingiliz

**Affiliations:** 1 Center for Infectiology, Berlin, Germany

Ogbuago and coworkers provide a small study on 19 HIV-HCV coinfected individuals that were treated with an 8-weeks course of sofosbuvir and ledipasvir. Overall, the SVR rate is 95% with only one individual not responding who was in-adherent. The study, although small, adds knowledge to the existing literature.

 Minor comments:

1. The authors should point out more clearly that they are dealing with a difficult-to-treat population here: A high percentage of AAs, high levels of IDU, and high BMI. This values the results even more.
2. In the Introduction the authors should point out that DAAs have changed treatment paradigms in HCV, but that 12 weeks treatment duration still proved to be the threshold hard to beat. It only worked so far with this regimen presented here. It will however change with new compounds.
3. In the discussion, the description of the ION-3 trial is slightly inaccurate: The non-inferiority of the 8-weeks regimen was an endpoint of the study. The 6 mil viral load threshold was a post-hoc-analysis.

I have read this submission. I believe that I have an appropriate level of expertise to confirm that it is of an acceptable scientific standard.

# Response

**Published:** 2017-06-21

**Authors:** Merceditas Villanueva

**Affiliations:** Yale University, USA

In response to Dr Ingiliz's comments:

1. It is correct that our cohort consisting of predominantly AAs, current or ex-IDUs, with high median BMI, and who are HIV co-infected are traditionally hard to treat populations so that it does make the treatment results all the more remarkable and in spite of the small sample size. This is now reflected in the discussion as follows:
  "The high SVR12 rate in our study is even more remarkable given that all patients were HIV infected and a significant proportion were African-American, had a high BMI and were active or current IDUs, all of which are characteristics of traditionally hard to treat populations. "
2. We have modified the sentence in the Introduction referencing 8-week and 12-week treatment regimens to suggest as accurately pointed out that for currently approved DAA regimens, 12-week treatment duration remains the standard for most patients, while 8 week regimens may be used for "selected cases". This is now reflected in the introduction as follows:
  "While 12-week DAA-based treatment regimens remain the standard treatment course for most HCV infected patients, emerging data suggests that 8-week rather than 12-week regimens may be effective for treatment among selected patients "
3. The paragraph has been rephrased to accurately reflect the primary results of the open label randomized ION-3 study as well as post hoc analysis as follows:
  "Kowdley *et al,* in a phase 3 open label randomized trial, showed that an 8-week treatment regimen of SOF/LDV resulted in a high SVR 12 rate among non-cirrhotic HCV infected individuals with genotype 1 infection that was non-inferior to an 8-week regimen with ribavirin or a 12-week regimen without ribavirin ^10^^ ^. In a post hoc analysis, lower relapse rates were observed among patients receiving 8 weeks of SOF/LDV who had baseline HCV RNA levels <6 million IU/ml (2%; 2 of 123)." 
